# Measuring *Daphnia* life history in the wild: The efficacy of individual field cages

**DOI:** 10.1002/ece3.8326

**Published:** 2021-11-26

**Authors:** Michael O’Connor, Daniel E. Sadler, Franziska S. Brunner, Alan Reynolds, Nicola White, Stephen Price, Stewart J. Plaistow

**Affiliations:** ^1^ Institute of Infection Veterinary & Ecological Sciences The University of Liverpool Liverpool UK

**Keywords:** *Daphnia*, field cage, field experiment, life history, multivariate phenotype

## Abstract

Life‐history studies are often conducted in a laboratory environment where it is easy to assay individual animals. However, factors such as temperature, photoperiod, and nutrition vary greatly between laboratory and field environments, making it difficult to compare results. Consequently, there is a need to study individual life histories in the field, but this is currently difficult in systems such as *Daphnia* where it is not possible to mark and track individual animals. Here, we present a proof of principle study showing that field cages are a reliable method for collecting individual‐level life‐history data in *Daphnia magna*. As a first step, we compared the life history of paired animals reared outside and inside cages to test the hypothesis that cages allow free flow of algal food resources. We then used a seminatural mesocosm setting to compare the performance of individual field cages versus glass jars refilled with mesocosm water each day. We found that cages did not inhibit food flow and that differences in life histories between three clones detected in the jar assays were also detectable using the much less labor‐intensive field cages. We conclude that field cages are a feasible approach for collecting individual‐level life‐history data in systems such as *Daphnia* where individual animals cannot be marked and tracked.

## Graphical abstract

There is a need to study individual life histories in the field, but this is currently difficult in systems such as Daphnia where it is not possible to mark and track individual animals. Here, we test the hypothesis that field cages are a useful method for collecting individual‐level life‐history data in *Daphnia magna*. We provide evidence that our cage design allows the collection of realistic, reliable, and repeatable measurements of life‐history data in the natural setting, as animals inside the cages experience the environment in the same way as those outside.

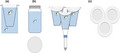

## 1 INTRODUCTION

Model organisms are an important part of experimental biology, especially in the field of evolutionary biology (Gasch et al., 2016), where taxa such as *Drosophila* spp., *Caenorhabditis elegans*, and *Daphnia* spp. allow the investigation and interpretation of some of evolution's biggest questions (Kellogg & Shaffer, 1993). Traditionally, work on model organisms has often been restricted to the laboratory (Barata et al., 2000). Laboratory experiments are useful for controlling natural environmental variation but also often result in individuals being studied in isolation (Kohler, 2002). This may exclude social aspects of the environment and other potentially important biological and environmental interactions (Morin, 1998). Since laboratory studies only capture a small part of the dynamic natural environment (Grodwohl et al., 2018), it can be difficult to know whether the results obtained in the laboratory are applicable to real‐world field conditions (Ieromina, 2014; Poorter, 2016).

*Daphnia* are a well‐established model organism used in a variety of studies across multiple fields including evolutionary biology, ecotoxicology, and genetics (Altshuler et al., 2011; Miner et al., 2012). *Daphnia's* short generation time, high fecundity, and clonality make it an ideal organism to carry out replicated experiments across multiple clonal lineages and environments (Ebert, 2005; Lampert, 2006). Furthermore, *Daphnia* are ecologically relevant; as a keystone species in many freshwater ecosystems, they act as both an algal grazer and as a prey species for a variety of aquatic predators (Ebert, 2005; Hebert, 1978; Lampert, 2006). Moreover, several *Daphnia* species have extensively mapped genomes accompanied by a plethora of genetic studies, allowing for understanding of life history and morphological responses and their evolution at a molecular level (Colbourne et al., 2005). However, much of our understanding of individual‐level *Daphnia* biology is based on the results of laboratory studies (Barata et al., 2000). Studies that take place in the field tend to be restricted to population‐level responses (Cabalzar et al., 2019; Zbinden et al., 2008). As a result, individual *Daphnia* life‐history responses in wild populations remain understudied (Bruijning et al., 2018; Burks et al., 2001).

A primary reason for this lack of individual‐based field studies, in both *Daphnia* and other aquatic model species, is the issue of replicated measurements of the same individuals. Difficulties associated with marking and tracking individuals for repeatable and reliable identification are magnified in the natural setting (Woodcock et al., 2016). Combined with the increased financial and logistical constraints associated with field work (Morin, 1998), this has led to a reliance on laboratory experimentation for aquatic invertebrates. The laboratory is a poor substitute for the dynamic natural environment. Dynamic variables in the wild such as temperature (Lagerspetz, 2006), photoperiod (Korpelainen, 1986), and nutrition (Ieromina et al., 2014) are often held constant in laboratory environments (Giebelhausen & Lampert, 2001). The lack of natural fluctuations affects the realism of laboratory studies, resulting in discrepancies, for example, in toxicity resistance between laboratory and field conditions in *Daphnia* (Duchet et al., 2010; Hatch & Burton, 1999) and other model organisms such as *Hyalella azteca* (Clark et al., 2015).

The need for individual‐level field studies goes beyond the limitations of laboratory realism. For the model organism *Daphnia*, questions related to its evolutionary ecology, for example, its ability to adapt to changing environments, can only be investigated under natural field conditions (Duchet et al., 2010; Yurista, 2001). Like most organisms, *Daphnia* are extremely phenotypically plastic, meaning that they change their phenotype in response to the environment that they are exposed to (Pigliucci, 2001; Plaistow & Collin, 2014; West‐Eberhard, 1989). Phenotypic plasticity is an integral part of responses to environmental change (Fox et al., 2019; Gienapp et al., 2008). It is therefore extremely important that experiments investigating the role of plasticity in evolution accurately reflect the natural environment that *Daphnia* are exposed to. Field cages represent a simple yet effective solution for studying phenotypic responses to changes in environmental conditions (Bjergager et al., 2012; O’Brien & Kettle, 1981). Small containers with mesh sides allow the monitoring of individual organisms while exposing them to many of the natural fluctuations in parameters such as temperature and food as experienced by the rest of the population. In this sense, cages can act as a midway point between the laboratory and the field (Bjergager et al., 2012). However, the design must ensure that the life‐history data collected is reflective of *Daphnia* living in a natural environment outside of the cage. Various field cage designs have been used in previous studies, both for cages holding individual *Daphnia* (Bjergager et al., 2012; Bruijning et al., 2018; Ieromina et al., 2014; Yurista, 2001) and for cages holding whole *Daphnia* populations (Haupt et al., 2009; Reichwaldt et al., 2004), yet the assumption that being inside the cage reflects natural conditions has, to our knowledge, never been tested.

In the present study, we wanted to address this lack of field cage validation and designed a field cage that allowed individual *D*. *magna* life‐history data to be collected in the context of a natural environment. First, in a laboratory experiment, we tested the hypothesis that cages allow free flow of algal food resources by comparing the life history of paired animals reared inside and outside cages. We then conducted a second experiment in a seminatural mesocosm setting where we compared the performance of individual field cages to the performance of assays conducted in glass jars that were refilled with mesocosm water each day, using three clones collected from the same population. We hypothesized that if cages performed adequately, clonal difference in life histories detected in jars would also be detected in cages.

## 2 METHODS

All *Daphnia magna* used were taken from laboratory lines isolated from Brown Moss Nature Reserve (52°57'01.2"N 2°39'05.6"W). *D*. *magna* were kept under standard conditions at 21°C and a 14:10 light:dark photoperiod for two generations in the laboratory before the experiment to reduce any potential maternal effects (Plaistow & Collin, 2014; Plaistow et al., 2015). The offspring from the second F3 clutch was then used for experimentation. Individuals in the laboratory experiment were fed ad libitum once daily on a high concentration food diet of 200 cells per µL of the algae *Chlorella vulgaris*.

### 2.1 Experiment 1: Comparing the life histories of animals inside and outside field cages

**FIGURE 1:**
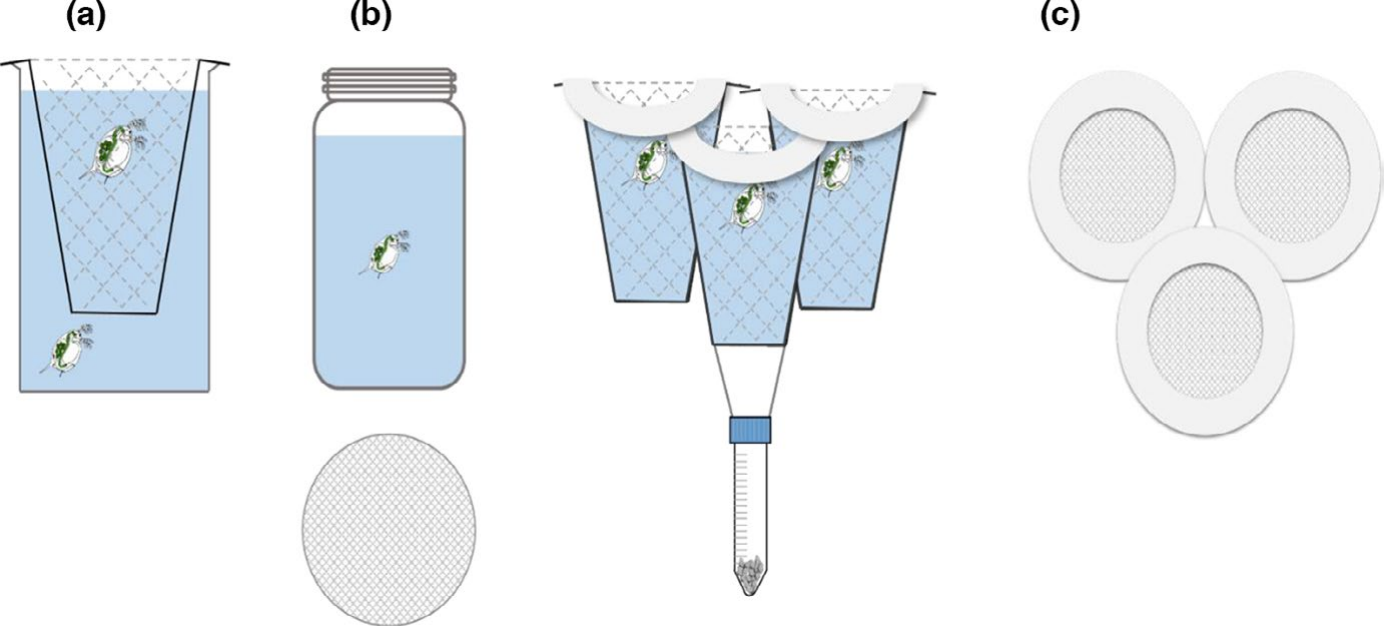
(a) The field cage design used in the initial laboratory experiment consisting of a Finum Brewing Basket M coffee filter within a glass beaker, with one *Daphnia* within the cage and one outside. (b) The jars used in the field experiment, with a secured mesh gauze on top, as displayed under the jar. (c) A set of three cages were tied together for the field experiment, with each containing one *Daphnia*, so each set of three contained a replicate from each clone. The field cages were attached to a falcon tube containing gravel as a weight. Each cage was secured with a mesh lid surrounded by polystyrene floats

The first experiment took place in the laboratory, under controlled conditions of 21°C and a 14:10 light:dark photoperiod using a single *D*. *magna* clone from Brown Moss. Forty‐three paired replicates were set up in caged and uncaged treatments, split over two time blocks, for a total of 86 individuals. We used Finum^®^ permanent filters (model Brewing Basket M, Finum, UK) as individual field cages. Each cage consisted of a plastic cup‐shaped frame (length 7.3 cm, diameter 6 cm) with sides and base composed of a 170‐μm stainless steel mesh (Figure 1). Forty‐three cages were placed inside 300‐ml glass jars filled with 260 ml of artificial pond water and enriched with 1.2 ml algal extract (Baird et al., 1989). A single *D*. *magna* neonate was placed inside the mesh cage with another neonate placed outside the cage such that each had access to roughly equivalent volumes of media in the jar (see Figure 1). The fine mesh size prevented neonate *Daphnia* from passing through, hand‐removing neonates from the inside of the cage with a pipette was similar to removing them from a glass jar. Each jar was filled with algal food (*Chlorella vulgaris*, 200 cells/µl) pipetted inside the cage and swirled gently to allow homogenization of the medium. Experimental containers were exchanged every other day to prevent build‐up of algae along the bases of the cage and beaker. Life‐history data were collected until individuals had dropped their second clutch as described in Plaistow and Collin (2014). Each animal was checked daily and photographed within 24 h after being born, upon reaching sexual maturity (assessed as the first time eggs appeared in the brood pouch), and after dropping their second clutch using a Canon EOS 350D digital camera connected to a Leica MZ6 dissecting microscope at 2.5× magnification. All images were then measured using the software ImageJ (Schneider et al., 2012). This methodology allowed us to collect data on the following life‐history traits: length at maturity (mm), length at second clutch (mm), age at maturity (days), age at second clutch (days), mean fecundity (mean number of neonates produced in clutches one and two), average offspring size (mean length across five neonates from clutch one and five neonates from clutch two of each individual *D*. *magna*), juvenile growth rate ((length at maturity–length at neonate)/age at maturity), and adult growth rate ((length at second clutch–length at maturity)/(age at second clutch–age at maturity)).

The data were analyzed using R version 4.0.2 (R Core Team, 2020). Any differences in the multivariate phenotype of caged and uncaged individuals were tested for using a permutational multivariate analysis of variance (perMANOVA). We calculated pairwise Gower distances using vegdist {vegan} to account for differences in scales between the life‐history variables (Gower, 1971). The calculated distance matrices were then used in perMANOVAs run for 9999 permutations using the adonis function {vegan} (Oksanen et al., 2013). Principal component analysis (PCA) was used to visualize the multivariate phenotypes using the prcomp function. Ellipses within the PCA plots show 95% percent confidence intervals around the centroids of the treatment groups. Life‐history traits were subsequently investigated individually using linear mixed models (LMMs), including the experimental block as a random factor (*Life history trait~Cage treatment*+*(1|block)*, to evaluate whether potential differences between caged and uncaged *D*. *magna* were caused by strong effects in a few traits or weak effects in many. Residual distributions were evaluated and where the Shapiro–Wilk test showed non‐normal distributions, or the Levene's test indicated heteroscedasticity, boxcox transformations were performed on the data using powerTransform {car} (Fox & Weisberg, 2011). A chi‐squared test was used to test for differences in mortality between caged and uncaged treatments.

### 2.2 Experiment 2: Comparing the performance of cages and jar assays in the field

The second experiment took place in a circular mesocosm of 2m diameter and 1m water depth at Ness Botanic Gardens (53°16'19.56"N, 3°2'44.16"W) in March 2019, during which time water temperature fluctuated around 10°C ± 2.5°C and photoperiod increased from 11.5h to 12.8h. We compared the life histories of three *D*. *magna* clones (BMH175, BMH47, and BMH 30) using two different methods: our individual field cages and a normal glass jar assay similar to that used in the laboratory, where the water in each jar was replaced on a daily basis. To set up the experiment, 10 replicates of each clone were added to individual jars and cages resulting in 60 individuals in total (see Figure 1). Each cage was attached to a polystyrene ring, allowing the cages to float at the surface of the mesocosm (see Figure 1b). Cages were attached in groups of three, with one replicate from each clone forming part of the trio. Each trio of floats was then weighted using a falcon tube filled with gravel, to prevent strong winds from capsizing the experimental containers (Figure 1c). Each jar was filled with 150 ml of mesocosm water and placed on a submerged plastic bench such that jars sat at roughly the same height in the water column as the field cages (Figure 1b). This ensured that temperature was consistent across both treatments. The jars were filled daily using water from the mesocosm (filtered through the mesh lid to prevent predators from entering). All experimental containers were covered with a fine mesh (300 µm) held by a durable elastic band to prevent escape should the cage/jar capsize. These mesh lids also served to prevent airborne predators or resting eggs from entering the containers. Growth data for both jar and caged *D*. *magna* were recorded until they reached maturity, collected by photographing each *D*. *magna* daily using a GXM‐HD51 digital microscope at 2.5× magnification and measuring the images on ImageJ (Schneider et al., 2012). As a result, we compared the juvenile growth rate, size at maturity, and age at maturity of the three different clones when reared in field cages, or in submerged glass jars filled with fresh media each day.

All statistics were performed in R version 4.0.2 (R Core Team, 2020). We fitted linear models (LMs) for each life‐history trait as a response variable and treatment (Cage, jar) and clone (BMH175, BMH47, and BMH 30) as fixed factors (Life history trait~Cage treatment*Clone). As above, any data observed to be non‐normal or heteroscedastic were boxcox transformed using powerTransform {car} (Fox & Weisberg, 2011). Mortality differences between experimental treatments were again tested for using a chi‐square test.

## 3 RESULTS

### 3.1 Experiment 1: Comparing the life histories of animals inside and outside field cages

**FIGURE 2:**
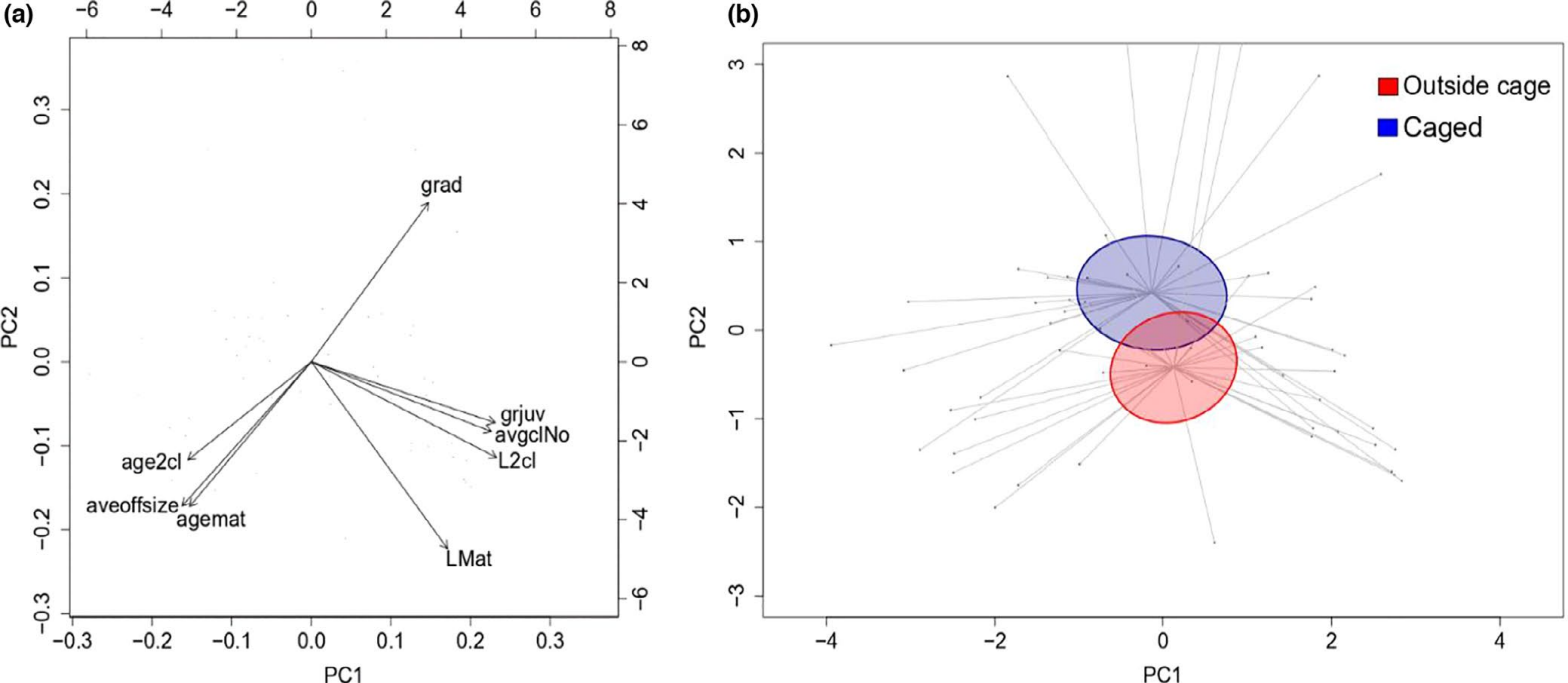
Principal component analysis of life‐history parameters across clones for the laboratory experiment. Contributions to principal component space are shown in the biplot (a) PC1 (41.77% of variation) vs PC2 (27.03%). The life‐history parameters measured are as follows: adult growth rate (grad), juvenile growth rate (grjuv), average clutch number (avgclNo), length at second clutch (L2cl), length at maturity (LMat), age at maturity (agemat), average offspring size (aveoffsize), and age at second clutch (age2cl). (b) 95% confidence intervals of group means are plotted for “Outside Cage” and “Inside Cage” *Daphnia*. Lines indicate distance of each individual from respective group centroids

There was a marginally significant difference in the multivariate phenotype of *Daphnia magna* individuals reared inside and outside of the cages (perMANOVA: *F*_1, 58_ = 1.746, *p* = .05, Figure 2b). The accompanying biplot revealed that *D*. *magna* within the cages tended to mature at a smaller size but grow more as an adult. This observation was confirmed by the univariate analysis which demonstrated that individuals reared inside the cages had a slower juvenile growth rate (LMM: *F*_1, 29_ = 8.468, *p* = .007), smaller size at maturity (LMM: *F*_1, 29_ = 13.91, *p* < .001), and produced slightly smaller offspring (LMM: *F*_1, 29_ = 6.794, *p* = .014). All other traits did not differ between treatments (LMM: *p* > .05; Table 1). There was no difference in mortality between caged and uncaged treatments (chi‐square: *χ* ^2^ = 0, df = 1, *p* = 1).

**TABLE 1:** Summary of individual life‐history trait analyses by ANOVAs for the laboratory experiment

LH trait	df	*F* value	*p* value
Age at maturity Lambda = 0.9290	1 29	1.8448	.1849
Size at maturity	1 29	13.907	.0008^***^
Juvenile growth rate	1 29	8.4675	.0068^**^
Adult growth rate	1 29	0.0778	.7823
Average clutch number	1 29	0.4291	.5128
Average offspring size	1 29	6.7941	.0143^*^

Note

To meet the assumptions of the ANOVA, boxcox transformations were performed where indicated by Shapiro–Wilk or Levene's test. Lambda values are listed for transformed variables.

* *p* < .05

** *p* < .01

*** *p* < .001.

**FIGURE 3:**
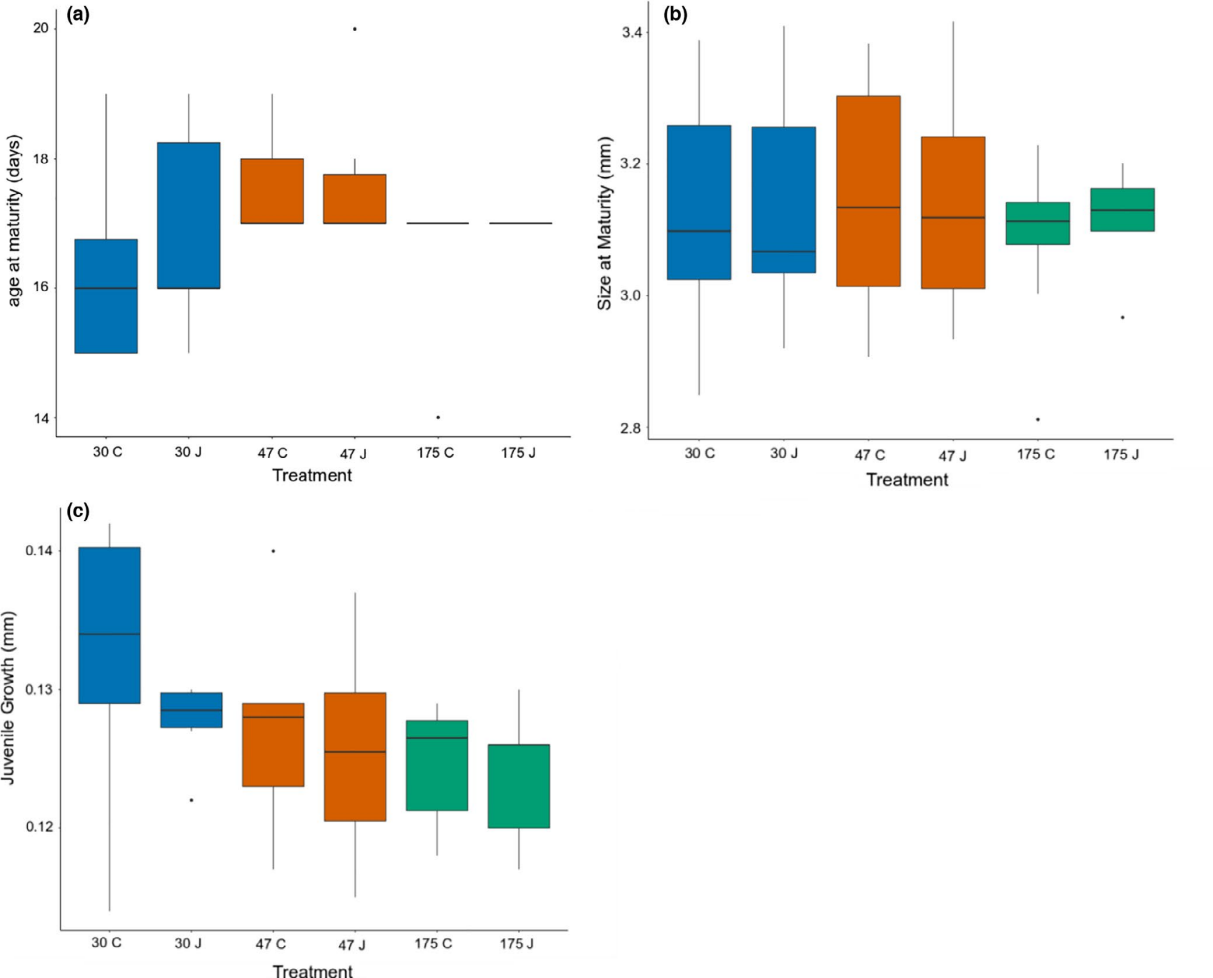
Life‐history traits; (a) age at maturity, (b) size at maturity, (c) juvenile growth rate, in *D*. *magna* of three clones (30, 47, 175) in two different containers; caged (C) and jar (J). Edges of the box represent the median, and the 25th and 75th percentiles, and the whiskers cover the 95th percentiles. Filled circles represent potential outliers

### 3.2 Experiment 2: Comparing the performance of cage and jar assays in the field

Being reared in a field cage or in a jar had no effect on the juvenile growth, size at maturity, and age at maturity across the three tested clones (LMs, all *p* > .3; Table 2). However, we did detect differences in the life histories of the three different clones which were independent of the cage treatment, specifically in juvenile growth rates (LM, Clone effect, *F*_2,36_ = 3.335, *p* = .047) (Figure 3). There was also a significant difference in *Daphnia* mortality between cage and jar treatments, with significantly more of the jar‐reared *Daphnia* dying before reaching maturity (chi‐square: *χ* ^2^ = 3.889, df = 1, *p* = .048).

**TABLE 2:** Summary of individual life‐history trait analyses by ANOVAs for the field experiment

LH trait	Treatment	df	*F* value	*p* value
Age at maturity Lambda = −0.2369	Cage type Clone Cage type: Clone Residuals	1 2 2 36	0.7829 2.6932 0.0866	.3821 .0813 .9172
Size at maturity	Cage type Clone Cage type: Clone Residuals	1 2 2 36	0.0471 0.2798 0.0344	.8294 .7576 .9663
Juvenile growth rate	Cage type Clone Cage type: Clone Residuals	1 2 2 36	1.0798 3.3349 0.3612	.3057 .0469^*^ .6993

Note

To meet the assumptions of the ANOVA, boxcox transformations were performed where indicated by Shapiro–Wilk or Levene's test. Lambda values are listed for transformed variables.

* *p* < .05.

## 4 DISCUSSION

*Daphnia* is an ideal model organism for investigating if and how shallow freshwater organisms can adapt to environmental change (Altshuler et al., 2011; Miner et al., 2012). However, attempts to study adaptation in *Daphnia* in the wild are hampered by the fact that it is not possible to mark and track individual animals. Field cages are a simple solution that may allow us to study individual‐level phenotypic response to environmental conditions *in situ* (Bjergager et al., 2012; Haupt et al., 2009; O’Brien & Kettle, 1981). Testing the assumption that animals inside field cages experience the environment in the same way as animals on the outside of the cages is an essential first step which is not addressed by most field cage using studies. In this study, we used a simple laboratory experiment to demonstrate that cages do not limit access to algal resources. Then, in a second experiment conducted in a mesocosm under seminatural conditions, we demonstrated that clonal variation in life histories was as detectable in field cages as it was in a much more labor‐intensive laboratory style assay conducted in the field. Juvenile survival was also improved in the field cages.

Model organisms have been incredibly useful for understanding many aspects of biology from gene functions to eco‐evolutionary dynamics. However, their value for understanding aspects of global change biology is limited if we cannot study them in the wild and quantify individual‐level responses to the real dynamic multifaceted cues of a natural environment. In some cases, we cannot study them in the wild because we know very little about their ecology (Parichy, 2015). But in other cases, such as *Daphnia*, the problem is simply that we cannot mark and track individuals. Field cages have previously been utilized as a way of getting around this problem (Bjergager et al., 2012; Bruijning et al., 2018; Ieromina et al., 2014; O’Brien & Kettle, 1981; Yurista, 2001). They have successfully been used to record life‐history data of population embedded individuals and small groups in the laboratory (Bruijning et al., 2018; Reichwaldt et al., 2004), seminatural (Bjergager et al., 2012), and natural environments (Haupt et al., 2009; Ieromina et al., 2014; Yurista, 2001) and to develop models explaining population‐level changes from individual life‐history parameters (Bruijning et al., 2018). O’Brien and Kettle (1981) used a dye experiment to demonstrate that media in and out of cages is quickly mixed and reported that the growth rate of populations kept in cages in the field were comparable to those of populations kept in the laboratory with excess food, although no data were presented. Furthermore, Reichwaldt et al. (2004) observed that algal growth rates were unchanged by daily addition and removal of fine mesh cages in their jars, but did not include *Daphnia* populations in and out of the cage treatment for comparison. None of the previous studies explicitly tested the assumption that animals reared inside field cages experience the environment in the same way as animals on the outside of the cages. And importantly, no study has ever previously tested the assumption that the cage mesh is fully permeable to food, which is key to individuals within cages experiencing the environmental conditions and provides individuals reared inside and outside cages with the same resource availability. Testing this assumption is critical if field cages are going to be a useful tool for understanding how individual‐level responses to environmental change scale up to the population, community, and ecosystem level (Bruijning et al., 2018; O’Brien & Kettle, 1981).

In our first experiment, we reared paired individuals inside and outside of field cages and fed algae on the inside of the field cage each day to test the hypothesis that animals reared outside the cage do not do significantly worse than animals on the inside where the food was placed each day. In fact, we found that individuals reared on the outside of cages actually did slightly better by growing faster, maturing at larger sizes, and producing slightly larger offspring. Although we cannot fully explain why animals on the outside did better, we suspect it could be because the volume of media on the outside of the cage (approx. 145 ml) was actually slightly greater than the volume of media inside the cage (approx. 115 ml), a result of the tapered shape of the cage. Irrespective of what caused the difference in the life histories of animals reared inside and outside cages, the fact that animals on the outside do not do worse than animals on the inside of cages where the food was put each day strongly supports the hypothesis that cages allow the free flow of algae. As a result, our findings support the idea that field cages could be used to quantify individual life histories in wild environments.

In order to test the hypothesis that field cages are useful for quantifying individual *Daphnia* life histories in the wild, we conducted a second experiment where we compared the life history of three *D*. *magna* clones from the same population using field cages and a typical laboratory style assay conducted in a seminatural mesocosm. Both approaches allowed us to detect differences in the life histories of the three clones that were the same irrespective of the method used. Moreover, there were no differences in the estimates of mean life‐history traits for each clone measured in jars and in field cages. Therefore, we can conclude that both methods were comparable in their ability to quantify clonal variation in individual level life histories. However, there are a number of reasons why the field cages are preferable to the jar approach. First, there is a significant difference in workload and required visits to the experimental site because jars must be changed every day and cannot be left in the wild for long periods of time. Second, changing jars every day increases disturbance and the requirement to handle and conceivably stress the *Daphnia*. This can also increase the risk of mortality; we observed that 13 *Daphnia* died in the jars but only 5 died in the cages. Third, *Daphnia* kept in jars might experience the same temperature variation and photoperiod, but the individuals being measured are still to some extent isolated from their environment and from dynamic changes occurring throughout the day in factors such as density cues, kairomones, and oxygen. For a jar assay, the dynamism of such cues is constrained by the frequency of the jar changes. While the lack of constant food flux through the day did not affect growth rates at early spring temperatures, nutrient limitation in closed containers is likely to have a greater impact in the summer when temperature‐dependent growth rates reach their maximum.

There are of course still important differences between our field cages and a truly wild setting. The main differences lie in the cages’ restriction of movement and associated behaviors such as diel vertical migration. While the cages can be left free to float across the entire surface of a mesocosm or pond, the *Daphnia* do not get to choose where they graze, nor do they get to move vertically in the ponds. Diel vertical migration could however be easily mimicked by moving the cages (even automatically) between depths in the morning and evening as done by Haupt et al. (2009) with larger population level cages, or alternatively by building cages as columns that allow free vertical movement. Furthermore, animals in cages are not directly exposed to predation which is often considered to be the strongest selection pressure operating in *Daphnia* populations (Lass & Spaak, 2003). However, individuals inside cages are exposed to predator cues and the large effect these have on individual life histories (Hammill et al., 2008), allowing researchers to separate the threat of predation from actual predation effects on populations in wild or semiwild conditions.

Demonstrating that field cages can be a useful and reliable way to measure individual *Daphnia* life histories in the field opens up a number of future possibilities. First of all, this opens up the possibility to study the differences between the laboratory and the field explicitly, for example, by comparing life histories of a large number of clones between the two settings and testing whether the extent of genotypic variation is comparable. Second, the cages will allow studying *Daphnia's* role in food webs and the wider community and its impact on ecosystem function in a more realistic way. Field cages will conceivably allow us to generate accurate individual‐level data required to parameterize models such as integral projection models (IPMs) that are used to predict population‐level responses to real environmental change from natural environments. Bruijning et al. (2018) have recently used such an approach to parameterize IPMs for laboratory populations of *Daphnia*, but no study has yet used such an approach in wild or semiwild populations. Finally, *Daphnia magna* is one of the most important ecotoxicology organisms. Although ecotoxicology is useful for defining acceptable doses of chemicals that can be released into the environment, this does not necessarily help us to understand the long‐term impact that exposures to novel anthropogenic stressors have in natural environments where populations are genetically variable and environments are dynamic. Field cages used in combination with replicated mesocosm studies could be one way forward (Bjergager et al., 2012; Ieromina et al., 2014; O’Brien & Kettle, 1981).

## 5 CONCLUSION

In summary, our ability to understand aspects of global change biology in model organism such as *Daphnia* is limited if we cannot study them in the wild and quantify individual‐level responses to natural environments. By demonstrating that individual‐level field cages do not limit access to resources and that cages are as capable of detecting clonal variation in life‐history traits as more labor‐intensive jar assays, our results demonstrate that field cages are a feasible approach for collecting individual life‐history data in natural environments. Having the capacity to measure genetic variation in responses to environmental cues in natural populations will, we hope, enhance the value of *Daphnia* studies aimed at predicting population‐level responses to environmental change.

## CONFLICT OF INTEREST

The authors declare that they have no conflict of interest.

## AUTHOR CONTRIBUTIONS

**Michael O'Connor:** Formal analysis (equal); Investigation (lead); Methodology (equal); Project administration (equal); Visualization (equal); Writing‐original draft (lead); Writing‐review & editing (equal). **Daniel E. Sadler:** Formal analysis (equal); Investigation (equal); Visualization (equal); Writing‐review & editing (equal). **Franziska S. Brunner:** Formal analysis (equal); Validation (equal); Writing‐review & editing (equal). **Alan Reynolds:** Formal analysis (supporting); Investigation (supporting). **Nicola White:** Investigation (supporting). **Stephen Price:** Investigation (supporting); Methodology (equal). **Stewart J. Plaistow:** Conceptualization (lead); Methodology (equal); Supervision (lead); Validation (equal); Writing‐review & editing (equal).

## Data Availability

Daphnia life‐history data for both laboratory and mesocosm experiments are publicly available on the Dryad Digital Repository https://doi.org/10.5061/dryad.zgmsbcccq
